# Laparoscopic cryoablation for small renal masses: Oncological outcomes at 5-year follow-up

**DOI:** 10.1080/2090598X.2020.1863308

**Published:** 2020-12-17

**Authors:** Michaël M.E.L. Henderickx, Annebeth E.C. Sträter-Ruiter, Alwine E. van der West, Harrie P. Beerlage, Patricia J. Zondervan, Brunolf W. Lagerveld

**Affiliations:** a Department of Urology, Amsterdam University Medical Centers (Amsterdam UMC), University of Amsterdam, Amsterdam, The Netherlands; b Department of Urology, Onze Lieve Vrouwe Gasthuis (OLVG), Amsterdam, The Netherlands

**Keywords:** Cryoablation, laparoscopy, small renal mass, focal therapy, oncological outcomes

## Abstract

**Objective**: To evaluate the oncological outcome at 5-year follow-up after laparoscopic cryoablation (LCA) for small renal masses (SRMs), as there is an increasing interest in ablative therapy for cT1a renal tumours due to the rising incidence of SRMs, the trend towards minimally invasive nephron-sparing treatments, and the ageing population.

**Patients and methods**: Between 2004 and 2015, 233 consecutive LCA were performed in 219 patients for SRMs at two referral centres. We only included those patients with ≥5 years of follow-up (*n* = 165) in a prospectively maintained database. A descriptive analysis was conducted for pre-, peri- and postoperative characteristics. A Kaplan–Meier analysis assessed overall (OS), disease-specific (DSS), and recurrence-free survival (RFS).

**Results**: The median (interquartile range [IQR]) age of our patient cohort was 68 (60.5–76) years. The median (IQR) body mass index was 26.2 (23.8–29) kg/m^2^, and the median (IQR) Charlson Comorbidity Index score corrected for age was 4 (2.5–6). The median (IQR) tumour diameter was 28 (21–33) mm. In all, 15% developed a complication in the first 30 days after LCA, of which 1% had a major complication (Clavien–Dindo Grade ≥III). The median (IQR) preoperative estimated glomerular filtration rate (eGFR) was 82.5 (65–93.75) mL/min/1.73 m^2^. The median eGFR decreased by 16.4% and 15.2% at the 3-month and 5-year follow-up, respectively. Persistence was found in 1%, local recurrence in 2%, and systemic progression in 4%. The OS, DSS, and RFS were 74%, 96.9% and 95.4%, respectively.

**Conclusion**: LCA is a safe and effective treatment for SRMs in selected cases and shows good oncological outcomes after 5 years of follow-up, with only 1% developing a major complication.

## Introduction

Partial nephrectomy (PN) is still the ‘gold standard’ for treating patients with small renal masses (SRMs) [1]. However, there is an increasing interest in ablative therapy for cT1a renal tumours, due to the increasing incidence of SRMs, the trend toward minimally invasive nephron-sparing treatments, and the ageing population [2].

Laparoscopic cryoablation (LCA) was first described by Gill *et al*. [3] in 1998. Today, the European Association of Urology (EAU) guidelines recommend focal therapy as a valid treatment option for cT1a renal tumours in elderly and comorbid patients who are unfit for surgery, patients with a genetic predisposition to develop renal tumours, and patients with bilateral tumours or with tumours in a (functional) solitary kidney with a high risk of complete loss of renal function following PN [1]. The AUA guidelines recommend physicians to consider focal therapy as an alternative to PN in the management of cT1a renal masses of <3 cm [4]. However, long-term oncological and functional outcomes are still scarce.

The present study evaluated the oncological and functional outcome at 5-year follow-up after LCA for SRMs (cT1a and cT1b).

## Patients and methods

### Study design and population

This study was a bicentric, non-randomised, retrospective analysis of a prospectively maintained database. Between 2004 and 2015, 233 consecutive LCAs were performed in 219 patients for SRMs at two referral centres [Amsterdam University Medical Centers (Amsterdam UMC) and Onze Lieve Vrouwe Gasthuis (OLVG)]. We only included patients with a ≥ 5-year follow-up or who died during this follow-up for final analysis. This resulted in 165 procedures for cT1 SRMs with ≥5 years of follow-up.

As both centres were referral centres, different treatment therapies were available during the study period (2004–2015) such as active surveillance, open or laparoscopic PN, and different ablative treatment modalities. All patients with RCC were discussed during a multidisciplinary oncological meeting resulting in a treatment proposition. Afterwards, a treatment technique was chosen in a shared-decision process with the patient.

### Surgical technique

All procedures were performed by three experienced surgeons through a transperitoneal or retroperitoneal access, as previously described by Beemster *et al*. [5]. After introduction of the trocars, the kidney was mobilised, followed by localisation of the tumour and imaging of the kidney and tumour with a steerable laparoscopic ultrasound (US) probe. After confirming and evaluating the tumour with US, a true-cut biopsy was taken for pathology in those cases with no preoperative pathology. The preoperative biopsy was taken in a separate session as described in a recent study by Widdershoven *et al*. [6]. The timing of the biopsy was discussed with the patient in a shared-decision manner. Preferably, the biopsy was advised to be taken preoperatively. For LCA the cryoprobes (Galil Medical®; Arden Hills, MN, USA) were positioned under laparoscopic and US guidance. The number and type of cryoprobes depended on the volume of the tumour. The freeze–thaw cycle was performed twice. Argon and helium gas were used for freezing and thawing cycles, respectively. After the last passive thaw cycle, an active thaw cycle helped in removing the cryoprobes easily. Haemostasis was achieved by compression with a haemostatic agent (Surgicel®, oxidised regenerated cellulose, Johnson & Johnson, New Brunswick, NJ, USA) or TachoSil® (sealant matrix, Takeda UK Ltd, Wooburn Green, High Wycombe, UK) on the insertion openings. When possible, Gerota’s fascia was closed over the tumour. Finally, the laparoscopic ports were extracted under vision.

### Follow-up

The follow-up protocol minimally consisted of a contrast-enhanced CT or MRI at 3, 6 and 12 months, and yearly thereafter up to a minimum of 5 years postoperatively (and afterwards at the discretion of the treating urologist). Additional imaging (contrast-enhanced US, CT or MRI) was performed at the urologist’s discretion when found to be necessary. All postoperative imaging for follow-up was interpreted by a board of certified abdominal radiologists with special expertise in focal therapy for kidney cancer. According to the international multidisciplinary Delphi consensus project by Zondervan *et al*. [7], presence of any radiological enhancement at the 3-month radiological follow-up was considered as persistence. A new (after a period of non-enhancement) enhancing or growing lesion, inside or in the margin of the ablated zone was considered local recurrence. Functional outcome was assessed with serum creatinine, measured 1 day before ablation and 1 day after, and at 3, 6 and 12 months, and yearly thereafter.

### Statistical analysis

A descriptive analysis was conducted for patient (age, sex, body mass index [BMI] and Charlson Comorbidity Index corrected for age [CCI-A] [8]) and tumour characteristics (size, side, clinical T-stage according to the 2017 version of the TNM classification [1], Preoperative Aspects and Dimensions for an Anatomical Classification [PADUA] score developed by Ficarra *et al*. [9]), and biopsy result (RCC and type, benign or non-diagnostic) and perioperative variables (number of cryoprobes, length of hospital stay, overall complication rate and complications according to the modified Clavien–Dindo Classification grading system [10]). Renal function preservation was calculated for the entire cohort as a percentage ratio of postoperative estimated GFR (eGFR) to preoperative eGFR [11].

The oncological outcomes (persistence, local recurrence, systemic progression, overall [OS], disease-specific [DSS], and local recurrence-free survival [RFS]) were analysed for patients with biopsy confirmed RCC (*n* = 131). Persistence was defined as residual tumour (contrast enhancement in the ablation site) on first imaging (at 3 months), whereas local recurrence was defined as any contrast enhancement on follow-up imaging at the site of ablation after initial imaging without contrast enhancement [7]. The OS was defined as the duration from date of LCA to death or 5-year follow-up, with no restriction on the cause of death. The DSS was defined as the duration from LCA until death due to RCC, and RFS was defined as the duration from the date of LCA until residual/recurrent tumour on imaging on follow-up imaging.

The Statistical Package for the Social Sciences (SPSS®) version 26 (IBM Corp., Armonk, NY, USA) was used to perform the statistical analysis. Medians and interquartile ranges (IQRs) were reported for continues variables. Frequencies were reported for categorical variables. A Kaplan–Meier analysis and plot assessed OS, DSS, and RFS.

## Results

The patients’ and tumour characteristics are shown in Table 1. The median (IQR) age of our patient cohort was 68 (60.5–76) years and comprised 72% men and 28% women. The median (IQR) BMI was 26.2 (23.8–29) kg/m^2^ and the median (IQR) CCI-A score was 4 (2.5–6). The median (IQR) tumour diameter was 28 (21–33) mm, meaning that 96% of the tumours were cT1a and 4% cT1b. According to the PADUA score, 45% had a low (6–7) score, 41% had intermediate (8–9) scores, and 14% had high (≥10) scores for anatomical complexity. Biopsy of the renal mass determined RCC in 79%, benign pathology in 17%, and a non-diagnostic result in 4%. Further analysis revealed 69% clear cell RCC, 17% papillary RCC type 1, 4.5% papillary RCC type 2, 9% chromophobe RCC, and 0.5% was not further specified.

**Table 1.** Pre- and perioperative characteristics

Variable	Value
Age, years, median (IQR)	68 (60.5–76)
Sex, %	
Male	72
Female	28
CCI-A, median (IQR)	4 (2.5–6)
BMI, kg/m^2^, median (IQR)	26.2 (23.8–29)
Preoperative eGFR, mL/min/1.73 m^2^, median (IQR)	82.5 (65–93.75)
Preoperative chronic kidney disease stage, %	
1	34.8
2	47
3a	11.6
3b	6.1
4	0.6
5	0
Tumour size, mm, median (IQR)	28 (21–33)
Side, %	
Left	54
Right	46
Clinical T-stage, %	
cT1a	96
cT1b	4
PADUA score, %	
Low	45
Intermediate	41
High	14
Biopsy result, *n* (%)	
RCC	131 (79)
Clear cell	90 (69)
Papillary type 1	22 (17)
Papillary type 2	6 (4.5)
Chromophobe	12 (9)
Not specified	1 (0.5)
Non-diagnostic	6 (4)
Benign	28 (17)
Number of cryoprobes, median (IQR)	4 (3–4)
Operation time, min, mean (range)	179 (75–362)
Length of stay, days, median (IQR)	3 (2–5)
Overall complication, %	
No	85
Yes	15
Clavien–Dindo Grade, *n* (%)	
0	141 (85)
I	11 (7)
II	11 (7)
IIIa	0 (0)
IIIb	2 (1)
IVa	0 (0)
IVb	0 (0)
V	0 (0)

Table 1 also presents the perioperative variables. A median (IQR) of 4 (3–4) needles were used per procedure. The mean (range) operation time was 179 (75–362) min. The median (IQR) hospitalisation time was 3 (2–5) days. In all, 15% of the patients (*n* = 24) developed a complication in the first 30 days after LCA. Of these complications, 14% were minor, of which 11 patients (7%) developed a Clavien–Dindo Grade I complication and another 11 (7%) developed a Clavien–Dindo Grade II complication. The Clavien–Dindo Grade I complications included dizziness, ileus and malaise (four patients) that required no further action, transient cardiac complications (four) that required no further treatment after a consultation with the cardiologist, transient fever (two) and a haemoglobin drop (one), which both required no intervention. The Clavien–Dindo Grade II complications were fever (five patients) and haematoma (six), which respectively required antibiotics or transfusions. Only 1% (two patients) had a major complication (Clavien–Dindo Grade ≥III). In one patient, the proximal ureter was injured during surgery with postoperative urine leakage as a result. A JJ ureteric stent was placed (Clavien–Dindo Grade IIIb). After 6 weeks, a retrograde pyelogram showed no leakage and the JJ stent was removed. Another patient developed an acute myocardial infarction postoperatively that required an intervention (Clavien–Dindo Grade IIIb).

**Figure 1.**
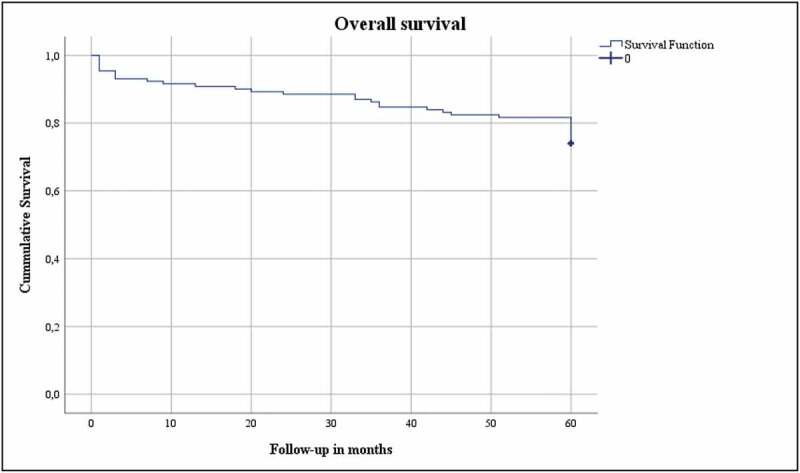
Overall survival

**Figure 2.**
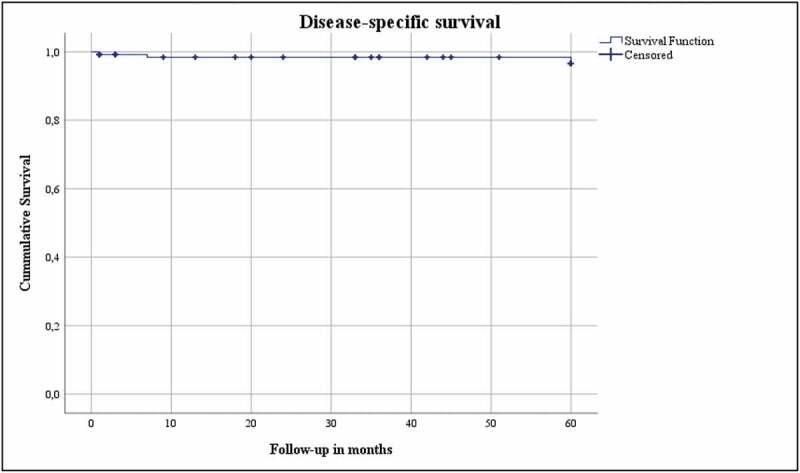
Disease-specific survival

**Figure 3.**
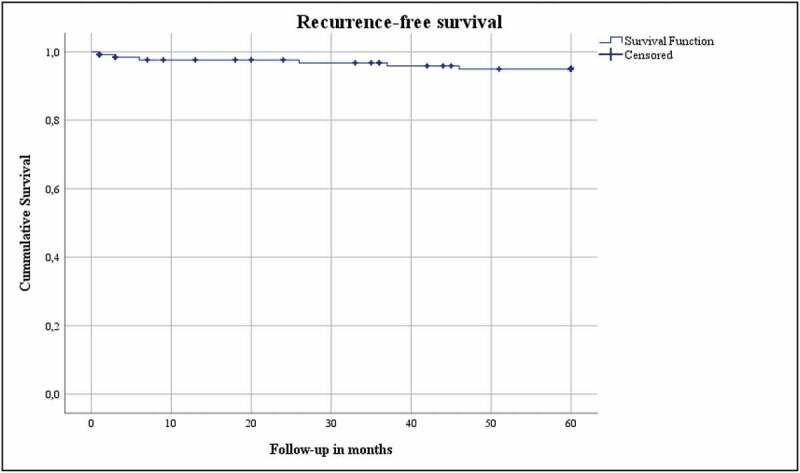
Recurrence-free survival

The functional and oncological outcomes are presented in Tables 1 and 2. The median (IQR) follow-up for both the entire cohort as for the biopsy confirmed RCC group was 60 (60–60) months. The median (IQR) preoperative eGFR 1 day prior to surgery was 82.5 (65–93.75) mL/min/1.73 m^2^. The median (IQR) eGFR at the 3-month and 5-year follow-ups was 69 (51–85) and 70 (49.75–86) mL/min/1.73 m^2^, respectively, which correlates to a 16.4% (13.5 mL/min/1.73 m^2^) increase after 3 months and 15.2% (12.5 mL/min/1.73 m^2^) after 5 years. Persistence was found in 1%, whereas local recurrence was seen in 2%, and 4% developed systemic progression. The OS, DSS and local RFS were 74%, 96.9% and 95.4%, respectively, and are shown in Kaplan–Meier plots (Figures 1–3). Finally, Table 3 gives details of the patients with persistence or local recurrence, the time of persistence/recurrence, and what treatment they received.

**Table 2.** Postoperative characteristics

Variable	Value
Persistence, %	1
Local recurrence, %	2
Systemic progression, %	4
Postoperative eGFR at 3 months, mL/min/1.73 m^2^, median (IQR)	69 (51–85)
Decrease compared to preoperative eGFR, %	16.4
Chronic kidney disease stage at 3 months, %	
1	18.4
2	46.8
3a	17.1
3b	10.8
4	7
5	0
Postoperative eGFR at 5 years, mL/min/1.73 m^2^, median (IQR)	70 (49.75–86)
Decrease compared to preoperative eGFR, %	15.2
Chronic kidney disease stage at 5 years, %	
1	20.6
2	43.7
3a	17.5
3b	10.3
4	5.6
5	2.4

**Table 3.** Follow-up treatment of the patients with persistence or local recurrence after LCA

Patient	Persistence or local recurrence	Time to persistence or local recurrence, months	Treatment
#1	Persistence	1	Active surveillance in systemic disease
#2	Persistence	3	Laparoscopic radical nephrectomy
#3	Local recurrence	6	Laparoscopic PCA
#4	Local recurrence	26	PN
#5	Local recurrence	37	PN
#6	Local recurrence	46	Laparoscopic PCA

## Discussion

The present study investigated the functional and oncological outcomes of LCA in a study population with a median age of 68 years and a median CCI-A of 4, with 5-year follow-up in a prospectively maintained database. The oncological outcomes were good at 5-year follow-up, with high DSS (96.9%) and RFS (95.4%) rates, and few treatment-related complications (1% Clavien–Dindo Grade ≥III).

The treatment options for SRMs range from PN (open, laparoscopic or robotic) over active surveillance to ablative therapies. The goal of the treatment should be to reduce cancer-specific mortality, to avoid progression of renal insufficiency to end-stage renal disease (ESRD) or dialysis, to avoid treatment-related complications, and to allow a good quality of life. Furthermore, the choice of treatment depends on tumour characteristics, clinical setting, and patient characteristics.

There is a variety of ablative techniques, e.g. CA, radiofrequency ablation, high-intensity focussed US, and microwave thermotherapy; however, CA is the most studied of these ablative techniques [12].

The preservation of renal parenchyma is one of the main advantages of CA, as the loss of renal parenchymal volume is closely related to the postoperative renal function [13,14]. Woldu *et al*. [15] state that when compared to PN, ablative therapy leads to less loss of renal parenchyma and thus less renal function loss. This is especially important in patients with solitary kidneys, chronic renal insufficiency, and elderly patients with comorbidities [13]. The eGFR in our present cohort showed a decrease of 16.4% and 15.2% at the 3-month and 5-year follow-up, respectively. Furthermore, only two patients in our present study population (1%) progressed to ESRD (eGFR <15 mL/min/1.73 m^2^) during follow-up. Zargar *et al*. [11] present a similar outcome for LCA in their study, with a decrease in eGFR of 18% at 39 months. Aron *et al*. [16] only found a reduction in eGFR of 10% in their study; however, this reduction was measured after 24 months. A recent European Registry for Renal Cryoablation (EuRECA) multicentre study on renal function loss in solitary kidneys by Sriprasad *et al*. [17] looked at the difference in pre- and postoperative eGFR. They found a clinically insignificant reduction in renal function of only 3.1 mL/min/1.73 m^2^ at 3 months after LCA. The decrease in kidney function cannot only be attributed to LCA, progressing age and comorbidities of the study population can also influence the declining eGFR [18].

The OS in our present study cohort was 74%, which can be explained by the characteristics of the study population. The lower OS could be due to a selection bias in the study population, where patients with comorbidities and/or unfit for surgery with a median CCI-A of 4 where chosen to undergo LCA. On the other hand, the DSS in the present study population was 96.9%. These results are consistent with the findings described in the literature. Aron *et al*. [16] described a 5-year OS of 84% and a DSS of 92%. Larcher *et al*. [19] found a somewhat higher OS of 95% and a DSS of 100% at their 5-year follow-up. However, they described a median time to death of >5 years (84 months) in their series. A large multicentre study reported an RFS of 90.4% at 5-year follow-up and an OS of 83.2% [20].

Finally, the local RFS was 95.4% in the present study cohort. This correlates with the RFS of 98% after 5 years described by Larcher *et al*. [19]. Caputo *et al*. [21] found a lower RFS at 5-year follow-up, namely 86.5%. However, the DSS (96.8%) and OS (79.1%) were comparable with our present findings. Nielsen *et al*. [20] published a large multicentre study in which they reported a DFS of 90.4% at 5-five-year follow-up and an OS of 83.2%.

In all, 15% of our present study population developed a complication in the first 30 days after LCA. Only 1% had major complications (Clavien–Dindo Grade ≥III). Nielsen *et al*. [19] reported similar results in their cohort of 808 patients, with an overall complication rate of 16.6% and severe complications in 3%.

Today, there is a trend towards even more minimally invasive techniques, namely percutaneous CA (PCA). The idea is that it is even less invasive and decreases morbidity, shortens hospitalisation, and leads to a quick recovery [22]. However, Schmit *et al*. [23] state that there is still a role for LCA if PCA is deemed to be difficult due to patient or tumour characteristics leading to a risk of treatment failure. Zargar *et al*. [11] describe that the advantages of LCA are the placement of probes under direct vision and the treatment of anterior tumours. Furthermore, no significant difference has been found between LCA and PCA for functional or oncological outcomes.

A limitation of the present study lies in the retrospective nature of the design and is therefore subject to the same bias as all other studies on this topic, as no prospective data exist [11]. Another major limitation is the lack of a control group to compare the results (e.g. patients undergoing PN). Finally, the lack of pathological confirmation of a local recurrence, as our oncological outcome was mainly based on radiological imaging and not on tumour biopsies.

## Conclusion

The present study found that LCA was a safe and effective treatment option for SRMs in selected cases. LCA offers good oncological outcomes at 5 years of follow-up, with high DSS (96.9%) and RFS (95.4%) rates, and few treatment-related complications (1% Clavien–Dindo Grade ≥III).
